# Diverse Macrophage Populations Contribute to the Inflammatory Microenvironment in Premalignant Lesions During Localized Invasion

**DOI:** 10.3389/fonc.2020.569985

**Published:** 2020-09-24

**Authors:** Ayman M. Ibrahim, Matthew A. Moss, Zane Gray, Michelle D. Rojo, Caitlin M. Burke, Kathryn L. Schwertfeger, Camila O. dos Santos, Heather L. Machado

**Affiliations:** ^1^Department of Biochemistry and Molecular Biology, Tulane School of Medicine, New Orleans, LA, United States; ^2^Tulane Cancer Center, Louisiana Cancer Research Consortium, New Orleans, LA, United States; ^3^Department of Zoology, Faculty of Science, Cairo University, Giza, Egypt; ^4^Donald and Barbara Zucker School of Medicine at Hofstra/Northwell, Hempstead, NY, United States; ^5^Department of Laboratory Medicine and Pathology, Masonic Cancer Center, and Center for Immunology, University of Minnesota, Minneapolis, MN, United States; ^6^Cold Spring Harbor Laboratory, Cold Spring Harbor, New York, NY, United States

**Keywords:** macrophage, microenvironment, scRNA sequencing, premalignancy, mouse model, localized invasion

## Abstract

Myeloid cell heterogeneity remains poorly studied in breast cancer, and particularly in premalignancy. Here, we used single cell RNA sequencing to characterize macrophage diversity in mouse pre-invasive lesions as compared to lesions undergoing localized invasion. Several subpopulations of macrophages with transcriptionally distinct profiles were identified, two of which resembled macrophages in the steady state. While all subpopulations expressed tumor-promoting genes, many of the populations expressed pro-inflammatory genes, differing from reports in tumor-associated macrophages. Gene profiles of the myeloid cells were similar between early and late stages of premalignancy, although expansion of some subpopulations occurred. These results unravel macrophage heterogeneity in early progression and may provide insight into early intervention strategies that target macrophages.

## Introduction

It is well-established that breast cancer progression occurs in a stepwise fashion beginning with hyperplasia, *in situ* carcinoma, invasive carcinoma, and ultimately progressing to metastatic disease (1). Accumulating evidence suggests that changes in the stromal microenvironment, including immune cells, play a central role in the initiation and progression of early stage disease (2). The microenvironment surrounding pre-invasive lesions is comprised of vasculature, myoepithelial cells, fibroblasts, extracellular matrix and immune cells, all of which interact with each other and premalignant cells to coordinate localized invasion and subsequent progression (3, 4). In particular, macrophages have been shown to have tumor-promoting roles in mouse models of early progression, where they are recruited to hyperplasias (5–7). Pro-tumorigenic functions of macrophages have made them attractive therapeutic targets, however, the mechanisms by which macrophages and other immune cells regulate early progression are poorly understood.

Macrophages exhibit an enormous amount of plasticity in both normal tissues and in cancer, and their function is largely dictated by their surrounding microenvironment. In the mouse mammary gland, macrophages are critical for proper ductal development and primarily function in tissue homeostasis (8). In cancer, monocyte-derived macrophages are recruited to tumors in a CCL2-dependent fashion where they are educated to promote tumorigenesis. Studies from mouse models have shown that distinct subpopulations of these tumor-associated macrophages (TAMs) function to promote angiogenesis, tumor cell invasion, immune suppression, as well as dissemination and growth at metastatic sites (9, 10). While myeloid cells including TAMs have been studied during the metastatic cascade, less is known about how macrophages function during localized invasion of premalignant lesions.

A number of recent studies have used single cell transcriptomics to define the immune microenvironment within tumors in various types of cancers, including breast (11–13). However, few studies have applied this approach to address the composition or functional role of macrophages in early ductal lesions, and particularly during the switch to invasive breast cancer. In this study, we utilized a p53^−/−^ mouse model of early breast cancer progression in which pre-invasive cells progress through ductal hyperplasia, low-grade mammary intraepithelial neoplasia (MIN) and high-grade MIN/invasive tumors in a predictable timecourse (14, 15). Single cell RNA-sequencing of CD45^+^ cells was performed to define unique populations of macrophages in premalignant lesions and lesions undergoing localized invasion. Our studies revealed several macrophage subpopulations that express genes common to both normal macrophages and TAMs, and highlight new gene signatures that define the premalignant niche.

## Materials and Methods

### Mice

BALB/cAnHsd (Balb/c) mice were purchased from Envigo. PN1a lesions were derived from *Trp53*^−/−^ mice (Balb/c) (16) and were maintained by serial transplantation into the cleared fat pads (#4 contralateral mammary glands) of 3 week-old female Balb/c mice as previously described (16, 17). Mice were housed in a pathogen-free facility under the NIH Guide for the Care and Use of Experimental Animals with approval from the Tulane School of Medicine Institutional Animal Care and Use Committee.

### Transplantation, Whole Mount Analysis and H&E Staining

For transplantation, mammary glands containing PN1a tissue were removed from donor mice at 8 weeks post-transplantation, minced into 1 mm fractions with a scalpel and re-transplanted into the cleared fat pads of 3 week-old female Balb/c mice as previously described (18). At 8 or 16 weeks post-transplantation, inguinal mammary glands containing PN1a outgrowths were fixed in cold 4% paraformaldehyde for 2 h and stained with carmine alum overnight (six mammary glands per timepoint). The next day, glands were dehydrated and imaged on a Leica M165 FC stereoscope (Leica Biosystems) as previously described (19). After imaging, mammary glands were embedded in paraffin, sectioned, and stained with hematoxylin and eosin (H&E) as previously described (20). H&E images were captured using an upright Nikon Eclipse microscope (Nikon Instruments). For tumors, mice were palpated twice weekly until tumors were measurable, and then measured three times a week. When tumors reached 1.2 cm in size, mice were euthanized and excised tumors were fixed with 4% PFA overnight and embedded in paraffin for subsequent immunostaining.

### Immune Cell Enrichment

Mammary glands containing PN1a lesions from 8 week (hyperplasia) and 16 week (high grade MIN with invasion) post-transplantation mice were excised with care to exclude the lymph node [four mice (eight mammary glands) per timepoint]. Glands were visualized under a Leica M165 FC stereoscope (Leica Biosystems) to confirm outgrowth. Then, mammary glands were pooled, minced and incubated in DMEM/F12 containing 2 mg/ml collagenase A (Roche) and 2 units of DNase (Sigma-Aldrich) at 37°C for 12 min with agitation (200 × rpm). Digested cells were neutralized with media containing 10% FBS, centrifuged at 450 × g for 5 min, and filtered through a 70 μm filter (BD Biosciences). The cell filtrate was then centrifuged at 450 × g for 7 min and the cell pellet was treated with ACK lysis buffer (Thermo Fisher) for red cell depletion, and neutralized with media containing 10% FBS. After centrifugation, single cells were resuspended in PBS containing 0.5% BSA/2 mM EDTA and incubated with mouse CD45 microbeads (Miltenyi Biotec) at 4°C for 15 min according to the manufacturer's protocol. Single CD45^+^ cells were enriched and purified as recommended by the manufacturer and prepared for sequencing.

### Single Cell RNA Sequencing

Five thousand individual cells with a viability of >88% was targeted for GEM generation and barcoding using 10x GemCode™Technology, which allows for partitioning thousands of cells into nanoliter-scale Gel Bead-In-Emulsions (GEMs), applying ~750,000 barcodes to separately index the transcriptome each cell. Full-length barcoded cDNA was generated and amplified by PCR, followed by enzymatic fragmentation, end-repair, A-tailing, and adaptor ligation. Single cell libraries were run using paired-end sequencing with single indexing with the NextSeq 550 platform. Data was collected as “.locs” files and downstream analysis was performed.

### scRNA-Seq Data Analysis

Single cell data (week 8 = 3,439 cells; week 16 = 4,412 cells) were aligned to mm10 using CellRanger v.3.1.0 (10x Genomics) (21), and downstream processing was performed using Seurat v3.1.1 (22). Cells with fewer than 250 features or higher than 10% mitochondrial gene content were removed prior to further analysis. Genes with fewer than three cells expressing then were removed, and the data were then log-normalized. Post-filtering analysis was performed on 3,075 cells (week 8) and 4,029 cells (week 16). Mitochondrial gene content and identifier count were regressed out. Principal component analysis was performed using the top 2,000 variable genes. This analysis was used to identify the number of significant components before clustering. Clustering was performed by calculating a shared nearest neighbor graph, using a resolution of 0.6. Subsetting into different cell types was performed using known markers for T-cells, myeloid cells, B cells and NK cells. Re-clustering was then performed using a similar method to that described above on each identified immune cell type. Myeloid cell re-clustering was based on expression of *Cd14* mRNA (23, 24), which included clusters 0, 2, 5, 6, 8, 9, 10, 11, and 12. Genes used to define each cluster (differentially expressed genes, DEGs) were determined using known cell type markers and using the FindAllMarkers function, which uses a Wilcoxon Rank Sum test to identify differentially expressed genes between all clusters in the dataset. Clusters 0, 2, 3, 4, 6, and 8 (**Figure 3**) were selected for DEG analysis across macrophage cell populations, and the top 20 DEGs are provided in Supplementary Table 1. Cell cycle scoring was performed using the CellCycleScoring function, using the gene lists provided by Seurat. Myeloid cell dendrograms were generated using the BuildClusterTree function in Seurat, using default arguments. Diffusion mapping was performed using the DiffusionMap function from the “destiny” R package (25). For analysis using the Immunologic Genome Project (Immgen) database, the top 20 genes in each cluster were analyzed for similarities to the indicated myeloid cell types using the My Geneset portal at immgen.org (26). Pathway analysis was performed using Enrichr (27). Gene ontology analysis was performed using the Gene Set Enrichment Analysis software (28, 29), on genes chosen using the FindMarkers function in Seurat. A complete list of genes utilized on each GSEA analysis are provided in Supplementary Table 2. Gene Ontology dot plots were generated using ggplot2 in R (29).

### Immunostaining

Paraffin embedded glands (*n* = 3) or tumors (*n* = 3) were cut into 5 μm sections, deparaffinized, rehydrated and subjected to antigen retrieval using 10 mM sodium citrate buffer. Following antigen retrieval, sections were blocked for 1 h in 7% donkey serum and stained with antibodies that detect Lyve-1 (1:60 R&D Systems, AF2125), CSFR1 (1:15 R&D Systems, AF3818), CD206 (1:1000, Abcam ab64693), and Gas6 (1:200 R&D Systems, AF986) at 4°C overnight. Slides were stained with Alexa Fluor-conjugated secondary antibodies (1:400; Thermo Fisher), mounted with ProLong™ Diamond Antifade Mountant (Thermo Fisher), and imaged on a Nikon Eclipse Ti2 confocal microscope with NIS Elements AR 5.20.02 software. For quantification, 10 random fields of view (FOV) were captured at 20X magnification for each mammary gland (three glands per timepoint), and the number of positive cells were counted. Two way ANOVA was used for statistical analysis when comparing the number of positive cells infiltrating within the lesion as compared to the number of positive cells surrounding the lesion.

## Results

### Single Cell Profiling of the Immune Microenvironment of Pre-invasive and Invasive Lesions

**Figure 1:**
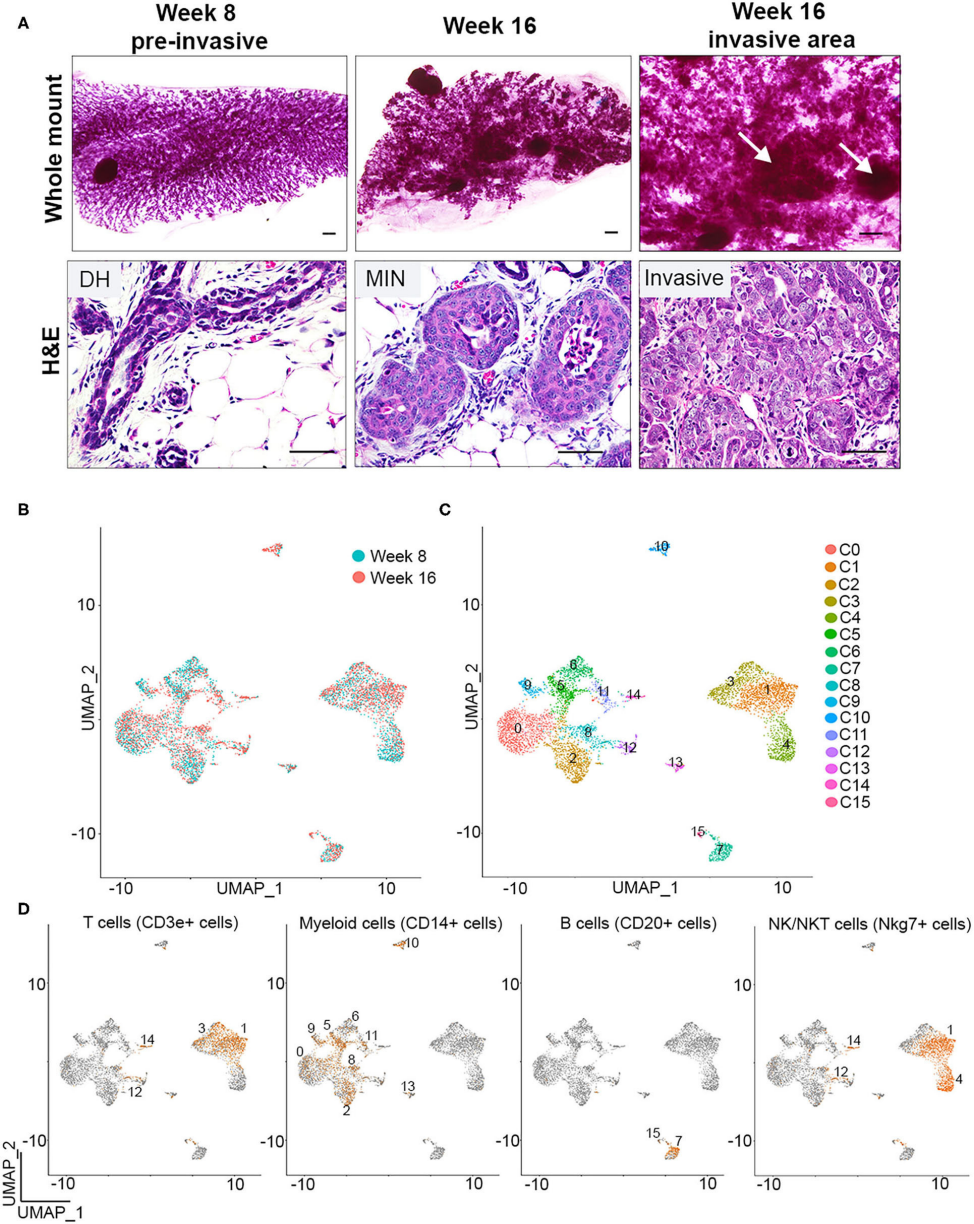
Single cell identification of immune cells during early breast cancer progression. **(A)** Whole mounts (top) and H&E staining (bottom) depicts PN1a outgrowths with ductal hyperplasia (DH) at 8 weeks (left), MIN (middle) at 16 weeks, or invasive lesions (right) at 16 weeks post-transplantation (*n* = 6). Arrows depict area of invasion. Scale bars = 200 μm (top) or 50μm (bottom). **(B)** UMAP distribution of CD45^+^ cells isolated from PN1a lesions at 8 weeks (blue) or 16 weeks (red) post-transplantation. **(C)** UMAP of CD45^+^ cells as 16 distinct clusters. **(D)** Feature plots depicting *Cd3e* (T cells), *Cd14* (myeloid cells) *Cd20* (B cells) and *Nkg7* (NK/NKT cells) mRNA expression.

We previously showed that macrophages are recruited to pre-invasive lesions with a high tumor-forming potential (PN1a) as compared to those that rarely form tumors (PN1b). In this model, p53^−/−^ hyperplastic cells are transplanted into the cleared fat pads of pre-pubertal Balb/c mice where they form ductal hyperplasias by 8 weeks post-transplantation, low grade MIN by 12 weeks, and progress to high grade MIN/invasive ductal carcinoma by 16 weeks (14, 16). We also showed that macrophages at the pre-invasive stage expressed a number of tumor-promoting cytokines and displayed pro-invasive phenotypes *ex vivo* (19). These studies were performed by co-culturing primary PN1a cells with bone marrow-derived macrophages (BMDMs), a model that may not recapitulate the diverse macrophage subpopulations localized to different regions of heterogeneous premalignant lesions. While these lesions are relatively homogeneous at 8 weeks post-transplantation, 16 week lesions consist of well-differentiated areas, poorly differentiated regions, as well as areas of invasion (Figure 1A). In the present study, we sought to identify and characterize potential macrophage diversity in these premalignant lesions.

To identify individual populations of macrophages during different stages of progression, CD45^+^ immune cells were isolated from PN1a lesions at 8 weeks (pre-invasive) or 16 weeks (invasive) post-transplantation, and single cell RNA sequencing (scRNA-seq) was performed using a 10x Genomics platform. Initial quality control analysis revealed the identification of ~2,000 genes per cell, yielded from an average of 10,000 reads, with an ~5% of these reads mapping to mitochondrial genes (Supplementary Figure 1A). Principal component analysis identified potential cell doublets or low quality cells, which were then removed from further analysis (Supplementary Figure 1B). An additional filtering step was employed after data clustering (UMAP), given the identification of outlier clusters with reduced number of cells (Supplementary Figure 1C). Post-filtering data clustering analysis demonstrated a similar distribution of cell clusters across week 8 and week 16, and distinguished 16 distinct subpopulations of CD45^+^ cells (Figures 1B,C). Further expression analysis of genes that define innate and adaptive immune cell lineages identified *Cd3e*-expressing cells (T cells, clusters 1 and 3), *Cd19* or *Cd20*-expressing cells (B cells, clusters 7 and 15), and *Nkg7*-expressing cells (NK/NKT cells, clusters 1 and 4). Myeloid cells were defined by *Cd14* expression and were present in nine separate clusters (clusters 0, 2, 5, 6, 8, 9, 10, 11, and 13) (Figures 1C,D, Supplementary Figures 1D,E) (23). When analyzing the abundance of cell lineages at each timepoint, the majority of the CD45^+^ cells were myeloid cells or T cells, both of which ratios increased in invasive lesions (16 weeks) as compared to pre-invasive (8 weeks) (Supplementary Figure 1F). Altogether, these data demonstrate the initial steps into a comprehensive identification of major immune populations during the progression from pre-invasive to invasive cancer.

### CD14-Expressing Cells Are Comprised of Monocytes, Macrophages, Dendritic Cells, and Other Myeloid Cells

**Figure 2:**
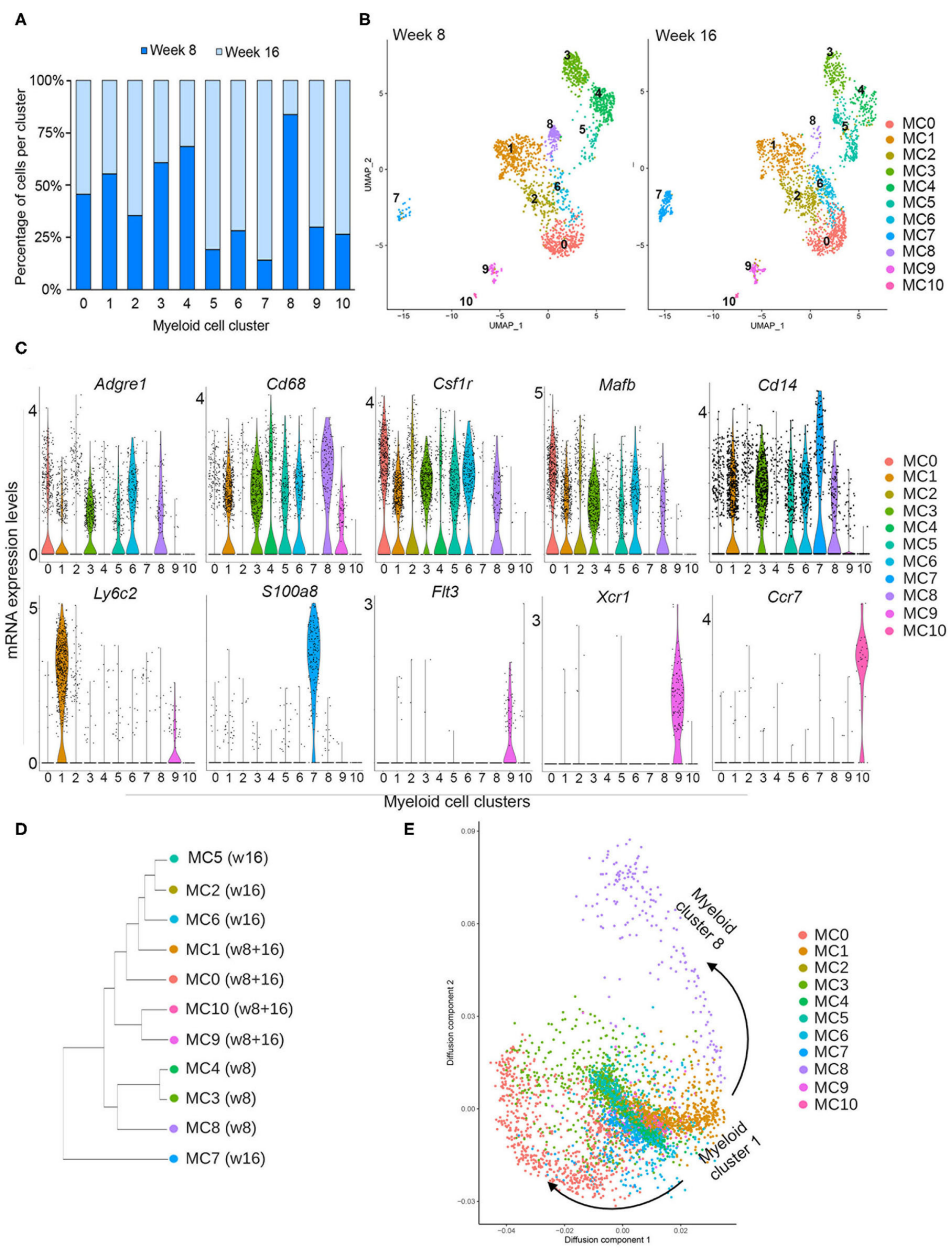
Myeloid subclusters based on CD14 expression. **(A)** Graph depicts the number of cells in each myeloid cell cluster as a percentage at week 8 (dark blue) and week 16 (light blue). **(B)** UMAPs depict the distribution of CD14^+^ myeloid cells at week 8 (left) or week 16 (right), identifying 11 distinct myeloid clusters (MC 0–10) and their abundance at each timepoint. **(C)** Violin plots demonstrate the distribution of various genes in each myeloid cluster that are commonly expressed in macrophages (*Adgre1, Cd68, Csfr1, Mafb, Cd14*), monocytes (*Ly6c2*), other myeloid cells (*S100a8*), and dendritic cells (*Flt3, Xcr1, Ccr7*). **(D)** Dendrogram shows similarities among subclusters and enrichment for each cluster at week 8 or week 16. **(E)** Diffusion map for myeloid subclusters demonstrating a branch point at cluster 1, which represents *Ly6c2*+ monocytes.

To distinguish different cell types in the myeloid lineage, unsupervised re-clustering of CD14^+^ immune cells was performed. As a result, *Cd14*-expressing myeloid cells were classified into 11 distinct populations (myeloid 0–10) (Figure 2A). Cell density analysis shows that clusters 3, 4, and 8 are more abundant in pre-invasive lesions (week 8), while clusters 2, 5, 6, 7, 9, and 10 are increased in number in invasive lesions (16 weeks) (Figure 2A). These data indicate that the abundance of subpopulations of myeloid cells varies across these two stages of progression. In addition, myeloid clusters 0 and 1 display similar numbers at weeks 8 and 16, suggesting that a fraction of myeloid cells remain unchanged during PN1a progression.

In order to survey the identity of the myeloid subclusters, we utilized a series of biased and unbiased gene expression tools (Figure 2B, Supplementary Figures 2, 3). The ImmGen Databrowser was used to preliminarily classify myeloid cluster cell types based on the top 20 differentially expressed genes (26). This analysis shows genes enriched for monocytes (MC1), macrophages (MC 0, 2, 3, 4, 6, 8), and dendritic cells (MC 9, 10), although cluster 2 appears to be constituted by a mixed population of cells. Myeloid cluster 5 is enriched for genes expressed by various cell types, and myeloid cluster 7 is enriched for both monocytic and granulocytic cell genes (Supplementary Figure 3). These suggestive cellular identities were further validated with analysis of genes known to delineate different myeloid lineages (Figure 2C). Myeloid clusters 0, 2, 3, 4, 5, 6, and 8 express general macrophage markers, such as *Adgre 1* (F4/80), *Cd68, Csfr1*, and *Mafb*, supporting a macrophage fate. Cells in cluster 1 highly express the monocyte marker *Ly6c2*, suggesting that they represent inflammatory monocytes. *Flt3, Xcr1*, and *Ccr7* are almost exclusively expressed by myeloid clusters 9 and 10, consistent with gene expression profiles associated with dendritic cells. Cluster 5 is highly enriched for proliferation genes such *MKi67, Pclaf* , and *Stmn1*, and additional analysis confirmed that these cells are primarily in G_2_/M of the cell cycle, suggesting that this subcluster represents a proliferative population (Supplementary Figures 2, 4A). While myeloid cluster 7 does not express macrophage markers, these cells highly express *Cd14, S100a8, Cxcr2, Il1b*, and *Cebpb* (Figure 2C, Supplementary Figure 4B), all of which have been reported in myeloid-derived suppressor cells (MDSCs) (30, 31). Additional functional assays are required to determine whether these cells are indeed MDSCs.

Total gene expression correlation analysis suggests that all myeloid cells broadly organize into three branches (Figure 2D). Cells predominantly present during week 8 (MC3, MC4, MC8) branch separately from those that are present, exclusively or not, at week 16 (MC1, MC2, MC5, MC6, MC9, MC10). The exception is myeloid cluster 7, which clusters separately from all subpopulations, suggesting a more distinct state for these cells. Further cellular diffusion analysis, which can predict cellular state transitions and potential developmental trajectories, shows that myeloid cells from cluster 1, which appear to be *Ly6c*^HI^ monocytes, may be related to all other clusters (Figure 2E). This analysis suggests that all of the myeloid subpopulations are related to cluster 1.

### Premalignant Lesions Contain Phenotypically Distinct Tissue Resident and Infiltrating Macrophages

**Figure 3:**
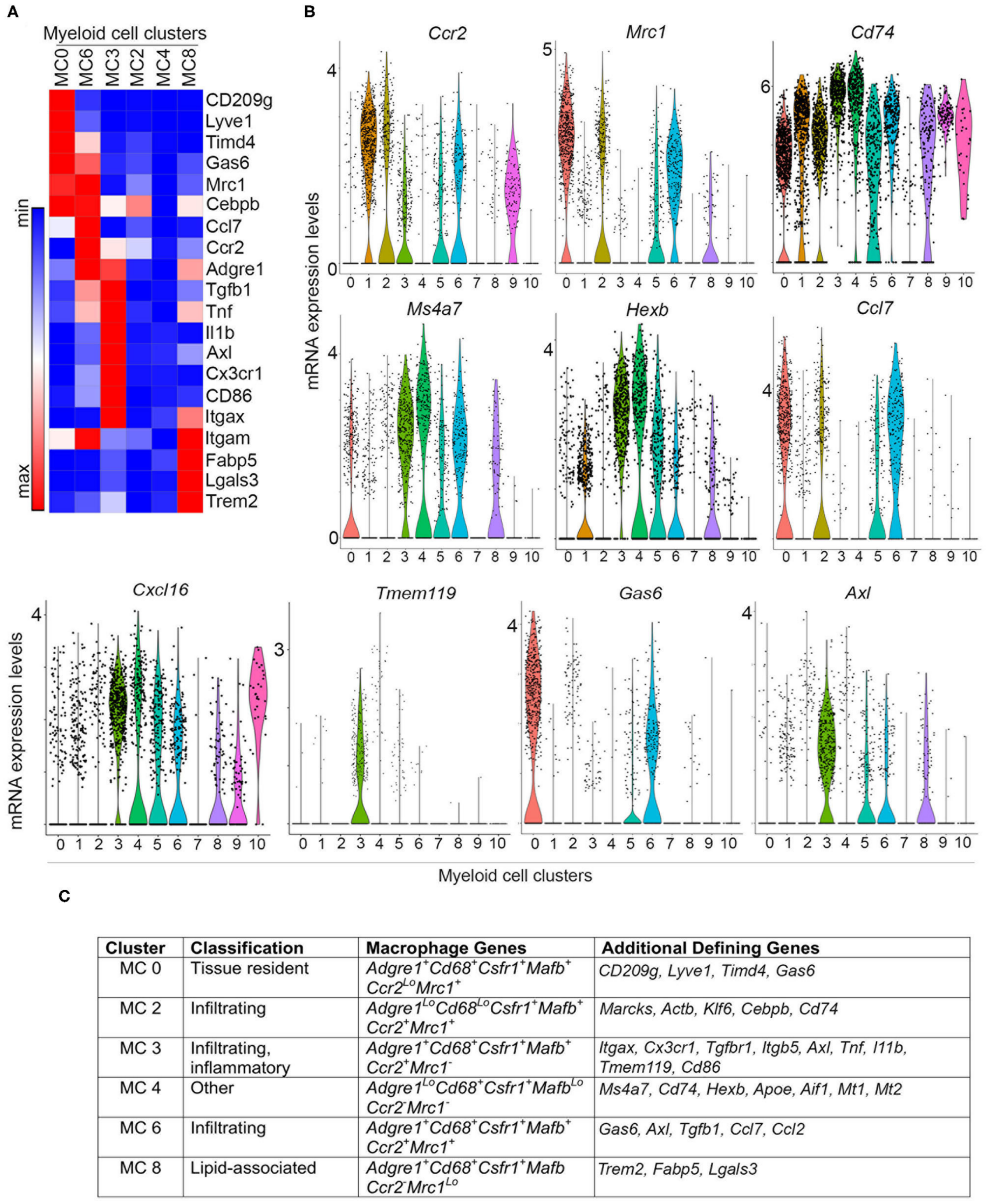
Differential gene expression in macrophage subpopulations. **(A)** Heatmap depicts differential gene expression of selected genes for macrophage clusters (MC 0, 2, 3, 4, 6, and 8). **(B)** Violin plots demonstrate the expression of selected genes across myeloid cell clusters. **(C)** Table summarizing the expression of macrophage genes and other defining genes identified by differential gene expression (Supplementary Figure 2, Supplementary Table 1), and putative classification of each macrophage subpopulation.

Myeloid clusters 0, 2, 3, 4, 6, and 8 express a number of genes characteristic of macrophages found in both normal mammary gland and mouse tumor models, suggesting that there are six putative macrophage subpopulations that respond to signals in developing PN1a lesions. To distinguish resident from infiltrating macrophages, we examined a set of differentially expressed genes among these clusters (Figures 3A,B). These genes were chosen based on the top 10 differentially expressed genes across all myeloid cells (Supplementary Figure 2), the top 20 differentially expressed genes amongst macrophage clusters (MC0, 2, 3, 4, 6, 8) (Supplementary Table 1), as well as some commonly reported markers in the literature. Myeloid cluster 0 highly expresses *Cd209g, Lyve1, Tim4d, Gas6*, and *Mrc1* (CD206), which have been shown to define a subset of tissue resident macrophages in the mammary gland and other tissues (32–35). *Ccr2* is expressed in myeloid clusters 2, 3, and 6, suggesting that these macrophages are recruited to the developing lesions (36, 37). Cluster 3 highly expresses *Itgax* (CD11c), *Cx3cr1*, and *Tmem119*, which have been described as ductal-associated macrophages in the normal mammary gland (38, 39). These cells are enriched for phagocytic genes such as *Axl* and *Hexb*, as well as genes that define pro-inflammatory macrophages (*Cd86, Tnf* ) and immunosuppressive function (*Il1b* and *Tgfb1*). Interestingly, cluster 3 also highly expresses *Cxcl16*, which was shown to define a subset of tumor-associated macrophages in Neu-driven mouse tumors characterized by *Cxc3r1* and *Mmp14* (40). Cluster 6 highly expresses tissue reparative/wound healing genes shared by MC0, such as *Mrc1* and *Gas6*, as well as pro-inflammatory genes common to MC3, including *Tnf, Ccl7*, and *Ccl2*. *Trem2, Fabp5*, and *Lgals3* are highly expressed in cluster 8, which have been shown to be enriched in lipid-associated macrophages (41). Interestingly, the macrophage populations lacked *Cd274* (PD-L1), which has been described in tumor-associated macrophages (TAMs) (42) (Supplementary Figure 5).

While some macrophage markers are expressed in myeloid clusters 2 and 4, these populations show weak expression for many of the genes analyzed. Upon further examination of the top 10 differentially expressed genes, cluster 2 highly expresses *Marcks, Klf6*, and *Actb*, all of which regulate cell motility and can modulate inflammation by mediating monocyte migration to inflammatory sites (Figure 3C, Supplementary Figure 2, Supplementary Table 1) (43, 44). High expression of *Cebpb* and low expression of *Adgre1* suggests that these cells are not fully differentiated, and may represent infiltrating monocytes transitioning into macrophages. Notably, cluster 2 expresses *Cd74*, which associates with MHCII during antigen presentation (45), and Ccl7, which is involved in monocyte and macrophage recruitment and chemotaxis (Figure 3, Supplementary Figure 5) (46). Myeloid cluster 4 expresses *Cd68* and *Csfr1*, but weakly expresses (or lacks) *Adgre1, Mafb, Ccr2*, and *Mrc1* (Figures 2B, 3). This population is enriched in genes involved in antigen presentation, such as *Cd74* and *Aif1*, as well as the lysosomal protease *Hexb*. Interestingly, the melatonin receptors Mt1 and Mt2 are also highly expressed in cluster 4, which have been shown to inhibit LPS-induced macrophage polarization *in vitro* (47) (Figure 3C, Supplementary Figure 2). Figure 3C summarizes these findings and lists potential defining genes for each macrophage subset.

**Figure 4:**
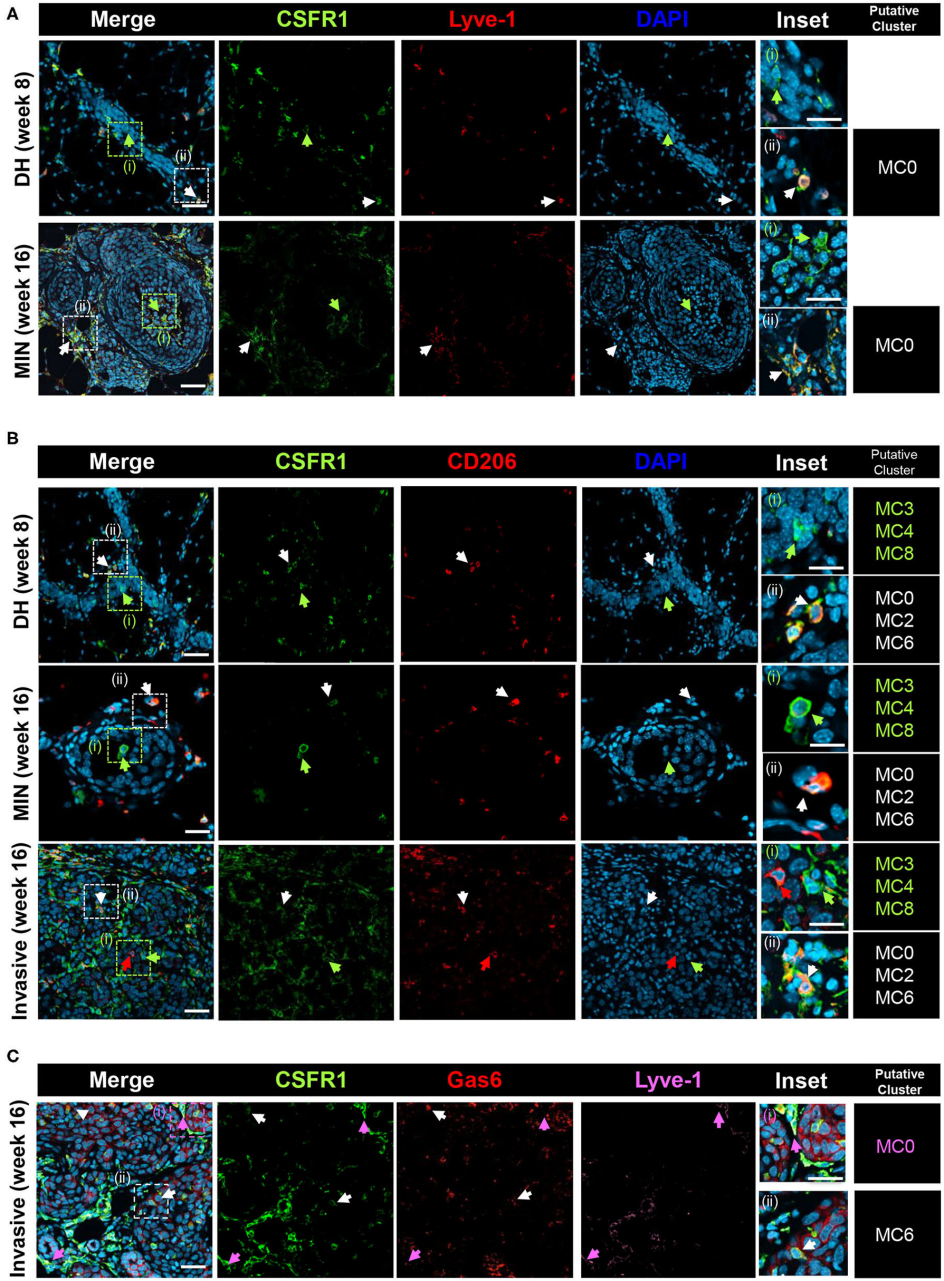
Co-localization of putative macrophage populations. PN1a lesions showing ductal hyperplasia (DH) (week 8), MIN (week 16) or areas of invasion (16 week) were stained with various antibodies and DAPI and imaged by confocal microscopy. Putative myeloid cell clusters representative of the staining are listed (far right column) **(A)** CSFR1 (green) or Lyve-1 (red). White arrows: CSFR1^+^Lyve-1^+^ (MC0); green arrows: CSFR1^+^Lve-1^−^. **(B)** CSFR1 (green) or CD206 (red). White arrows: CSFR1^+^CD206^+^ (MC0, MC2, MC6); green arrows: CSFR1^+^CD206^−^(MC3, MC4, MC8). **(C)** CSFR1 (green), Gas6 (red) or Lyve-1 (purple). White arrows: CSFR1^+^Gas6^+^Lyve-1^−^(MC6); purple arrows: CSFR1^+^Gas6^+^Lyve-1^+^(MC0). Scale bars = 100 and 25 μm for inset (*n* = 3 per timepoint).

To gain further insight on the macrophage subpopulations, immunostaining was performed on PN1a lesions at different stages of progression, including established tumors (Figure 4, Supplementary Figure 6). In 8 and 16 week lesions, Lyve-1^+^CSFR1^+^ cells (MC0) reside in regions surrounding the lesions or in the stroma, whereas Lyve-1^−^CSFR1^+^ cells are also found within the lesions and intercalating between hyperplastic cells (Figure 4A, Supplementary Figure 6A). This finding is consistent with reports of stromal-associated Lyve-1^+^ tissue resident macrophages in the normal mammary gland (35). Cells found within the ductal cells (week 8) or MIN lesions (week 16) are predominantly CD206^−^CSFR1^+^, suggestive of myeloid clusters 3, 4, or 8 (Figure 4B, Supplementary Figure 6B). As these cells infiltrate into the lesions, potentially at regions of inflammation and necrosis, they are more likely cells from myeloid cluster 3 (*Ccr2*^+^), although additional markers are needed to confirm. CD206^+^CSFR1^+^ macrophages (MC0, MC2, MC6) are primarily located in areas surrounding the lesions in pre-invasive stages (DH week 8, MIN week 16), however are also present in areas of invasion and persist in established tumors. Notably, rare CD206^+^CSFR1^Lo^ cells are detected in invasive regions as well as established tumors (Figure 4B, Supplementary Figure 6C). Lastly, Lyve-1^−^Gas6^+^CSFR1^+^ cells, which likely represent cells in myeloid cluster 6, localize to invasive regions (Figure 4C, Supplementary Figure 6A). These results support the existence of tissue resident and recruited macrophages in early progression, though additional specific markers are required to validate each subpopulation.

### Macrophages in the Premalignant Microenvironment Are Defined by Pro-inflammatory and Tumor-Promoting Pathways

Our cellular identification approach and gene expression analysis suggest that there are six distinct macrophage subpopulations in premalignant PN1a mammary glands. Myeloid clusters 0 and 3 share genes found in macrophages in the normal mammary gland (35, 38, 39, 48), whereas clusters 2, 6, and 8 express genes that have been described in established tumors (37, 41, 49, 50). Cluster 8 decreases substantially in invasive lesions (Figure 2A), indicating that lipid-associated macrophages do not expand during progression to tumors in this model. In contrast, clusters 2 and 6 both increase substantially during localized invasion (16 weeks) as compared to pre-invasive stages (8 weeks) (Figure 2A). Thus, we focused our studies on further defining myeloid clusters 0, 2, 3, and 6.

**Figure 5:**
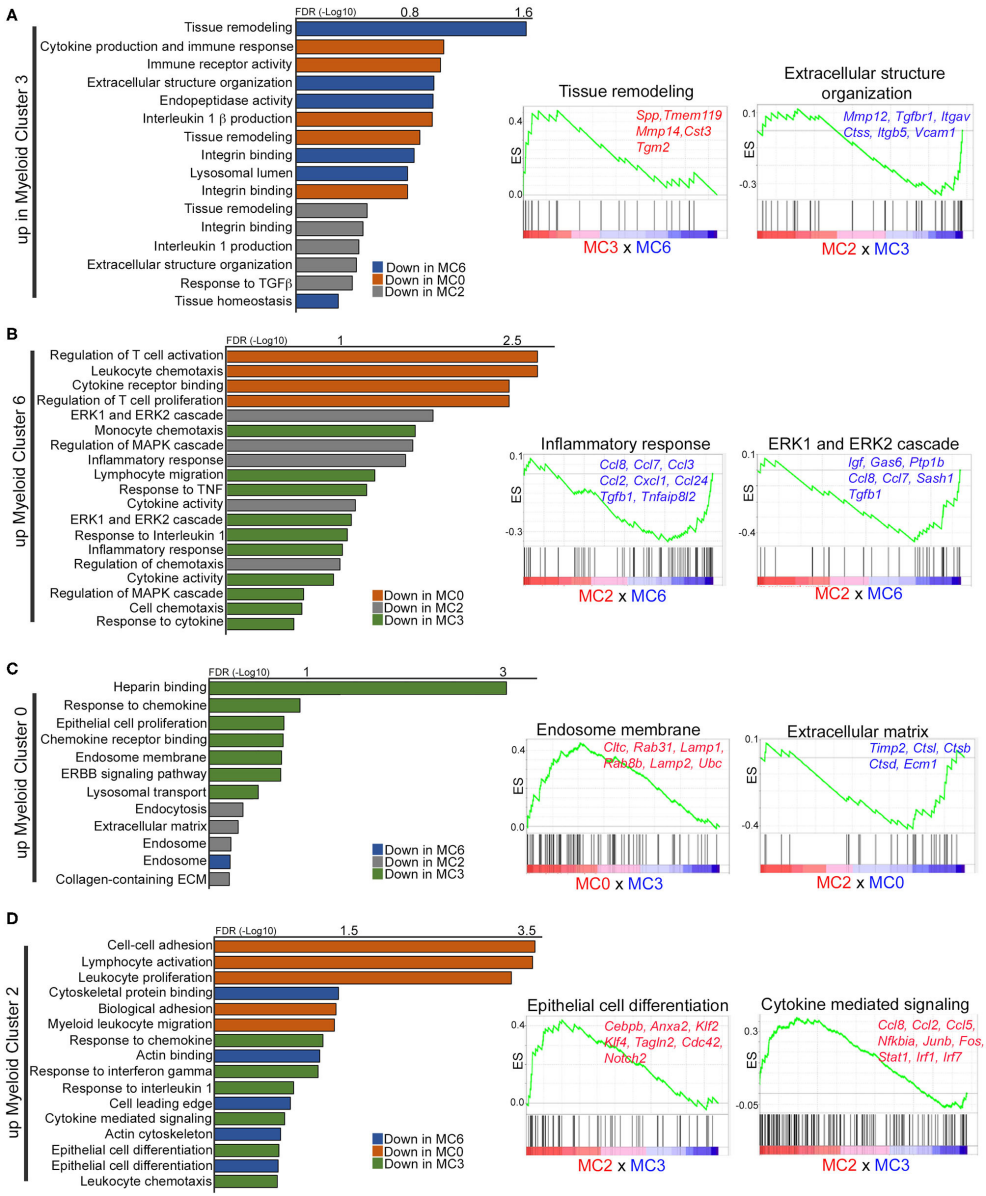
Gene set enrichment analysis identifies unique gene ontology in macrophage subpopulations. **(A–D)** Graphs and enrichment plots show significantly changed gene ontology (GO) terms from GSEA analysis of each macrophage cluster compared to each other (MC0 × MC3, MC0 × MC6, MC3 × MC6, MC2 × MC0, MC2 × MC3, MC2 × MC6). Graphs depict GO terms that are increased (FDR < 1) in myeloid clusters 3 **(A)**, 6 **(B)**, 0 **(C)**, or 2 **(D)**. Enrichment plots illustrate selected significantly upregulated GO terms with representative genes that are significant.

To gain insight on the function of these subpopulations, gene set enrichment analysis (GSEA) was used to identify pathways and ontology terms associated with differentially expressed genes across combinatorial analysis of myeloid clusters 0, 2, 3, and 6 (MC0 × MC3, MC0 × MC6, MC3 × MC6, MC2 × MC0, MC2 × MC3, MC2 × MC6). Gene ontology revealed that cluster 3 is enriched for pathways involved in tissue remodeling and integrin signaling, as well as Il-1β-mediated inflammation, as compared to other clusters (Figure 5, Supplementary Table 3). In particular, differentially expressed genes in cluster 3, including *Mmp12, Mmp14, Itgav, Pdgf*, and *Vcam1*, have been shown to modulate vascular remodeling (Figure 5A). Genes in these pathways are significantly downregulated in cluster 6, which in contrast are enriched for pathways involved in T cell activation, chemotaxis, and MAPK/ERK signaling (Figure 5B). Differentially expressed genes include numerous inflammatory chemokines that mediate macrophage recruitment (*Ccl2, Ccl3, Ccl7, Ccl8*) as well as genes that inhibit inflammation (*Gas6, Ptp1b, Igf* ) (5, 46, 49). While numerous pathways, such as T cell activation and proliferation, Leukocyte chemotaxis, and Response to TNF, implicate anti-tumor activity, ERK signaling in macrophages has been shown to be tumor-promoting by exerting both anti-inflammatory and pro-invasive properties (51). Cluster 2 upregulates genes involved in cell adhesion and the actin cytoskeleton, supporting the idea that these cells are infiltrating monocytes transitioning to macrophages. This subpopulation also differentially expresses a number of genes involved in cell growth and differentiation, such as *Anxa2, Notch2, Rpbj*, and *Myadm* (Figure 5D). These cells appear to contribute to inflammation through STAT/IRF/NFκB signaling. Finally, cluster 0 is enriched for pathways involving endocytosis, endosomes, and the ECM (Figure 5C), consistent with stromal-associated tissue resident macrophages in the mammary gland (35). This subpopulation differentially expresses genes that have been shown to be tissue-reparative (*Hmox1, Gas6*) (49, 52) and tumor-promoting (*Pf4, Fgfr1*, and *Nrp2*) (53–55).

Global gene expression analysis (Enrichr) was also performed to define enriched functional pathways across all myeloid clusters (27) (Supplementary Table 4). Myeloid cluster 3, which is enriched in pre-invasive lesions (week 8), is defined by pathways involved in focal adhesion signaling, based on the expression of a number of integrins, and matrix metalloproteinases (MMPs), consistent with a role in tissue remodeling. PPAR signaling, Retinol Metabolism and Glutathione Metabolism pathways are unique to cluster 8 in pre-invasive lesions, supporting the hypothesis that these cells are lipid-associated macrophages (41). Myeloid cluster 4 is enriched for *C1qb, C1qa* and *C1qc*, which have been shown to have anti-inflammatory properties in macrophages (56), and may suggest that complement genes drive the cluster. Interestingly, cluster 6 is uniquely characterized by Igf signaling, which has been shown to be active in alternatively activated macrophages (57). Altogether, these results demonstrate diverse subpopulations of macrophages, all of which appear to have tumor-promoting characteristics (10, 34, 58).

### Macrophages Are Characterized by Unique Pathways During Localized Invasion

**Figure 6:**
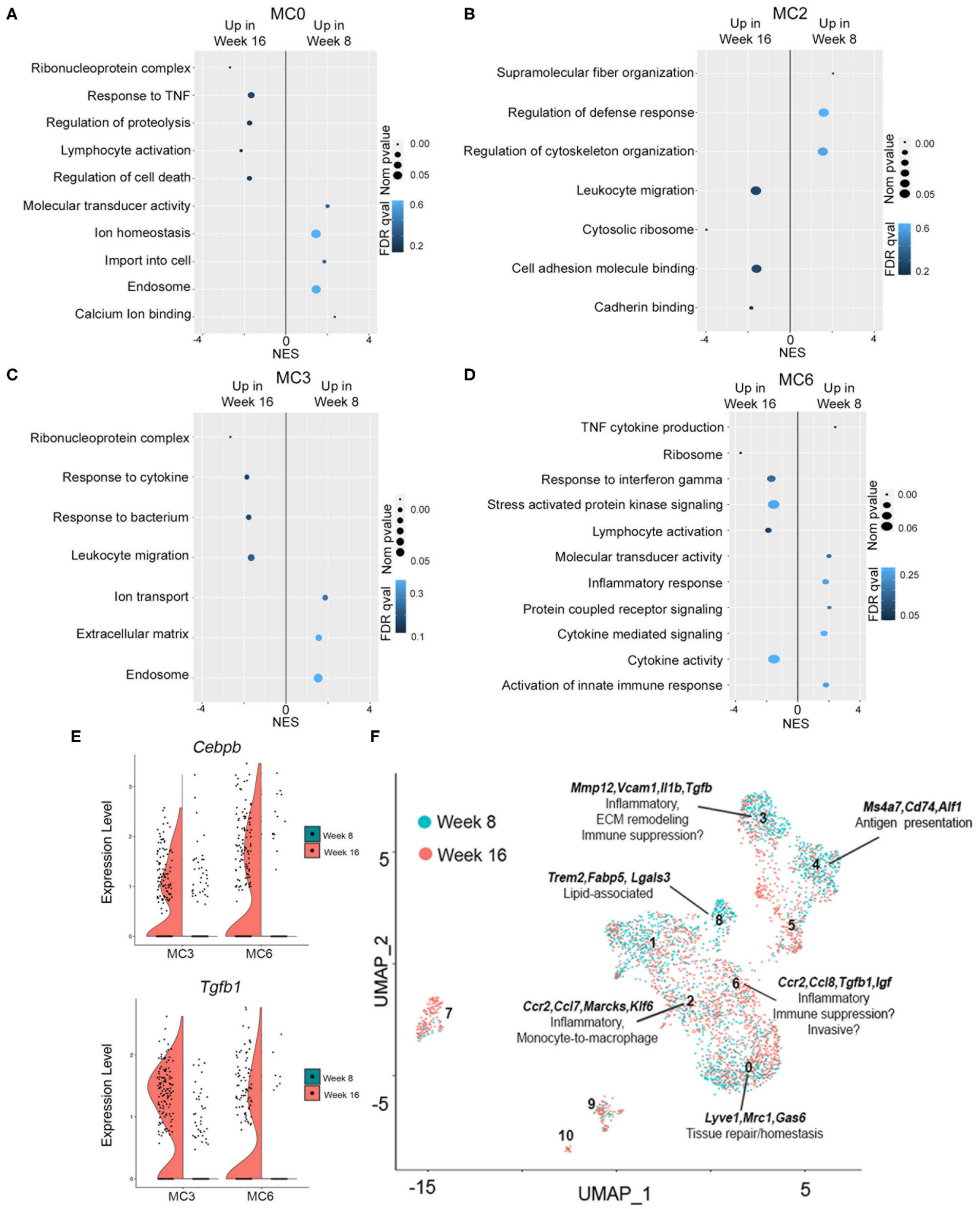
Gene expression in pre-invasive as compared invasive lesions. **(A–D)** Plots show *p*-value (<0.05), FDR (<1) and enrichment score (NES) for genes significantly upregulated at 16 or 8 weeks for myeloid clusters 0 **(A)**, 2 **(B)**, 3 **(C)**, or 6 **(D)**. **(E)** Violin plots demonstrating gene expression of *Cebpb* and *Tgfb1* at week 16 as compared to week 8 in myeloid clusters 3 and 6. **(F)** UMAP shows myeloid clusters 0–10 at week 8 (blue) and week 16 (red) of progression. Defining genes are displayed for each macrophage cluster, and potential functions are hypothesized.

We anticipated that we would observe vast plasticity amongst macrophages in pre-invasive lesions as compared to lesions undergoing localized invasion. While distinct macrophage subpopulations were identified during early progression, their gene expression profiles are strikingly similar at week 8 as compared to week 16 post-transplantation (Figures 2A, 6F).

To identify potential differences in gene expression in pre-invasive as compared to invasive lesions, analysis within the same cell populations but across pre-invasive and invasive time points (week 8 × week 16) was performed. Global pathway analysis (Enrichr) suggests that networks that mediate the immune response (Macrophage Markers, Inflammatory Response Pathway) are enriched in cell clusters more abundant at week 8, while genes associated with tumor-promoting pathways (MAPK signaling pathway, EGFR signaling pathway, TGFβ Signaling Pathway) are enriched at week 16 (Supplementary Table 5). Interestingly, pathway enrichment (GSEA) of differentially expressed genes across myeloid clusters 0, 2, 3, and 6 indicates a significant enrichment in genes involved in ribosomal biogenesis in invasive lesions as compared to pre-invasive (Figure 6, Supplementary Table 6), a process that has been shown to be hyperactivated during cancer initiation and progression (59–61). Gene ontology shows that cluster 0, putative tissue resident macrophages, are enriched for pathways involving calcium modulation and endocytosis at week 8, whereas cells present at week 16 upregulate genes involved in proteolysis and cell death (Figure 6A). Cells in myeloid cluster 2 show enrichment for genes involved in cytoskeleton organization and defense response at week 8, as compared to genes associated with cell adhesion and migration at week 16 (Figure 6B). Cluster 3 shows a significant increase in genes involved in ECM remodeling and endosome-associated pathways at week 8, while week 16 is defined by pathways involved in inflammation (Figure 6C). Lastly, week 16 cells in myeloid cluster 6 is dominated by genes involved in ribosomal biogenesis and translation followed by immune stimulatory pathways, while cells in week 8 are characterized by pathways involving the innate immune response (Figure 6D, Supplementary Table 6). Importantly, across all cells in myeloid clusters 3 and 6, *Cebpb* and *Tgfb* are both significantly upregulated in invasive lesions as compared to pre-invasive (Figure 6E). Given the role of C/EBPβ and TGFβ in mammary epithelial cells, breast cancer, and immune suppression (62–67), these findings suggest that these macrophage populations may have immunosuppressive function in established tumors.

## Discussion

Macrophage heterogeneity in cancer has emerged as an important factor in predicting outcome and response to therapy (12, 34). Macrophages are recruited to tumors where they are activated to exert pro-tumorigenic functions, and thus, targeting macrophages or reversing tumor-induced polarization has been pursued as a potential therapeutic strategy (68). While a number of studies have focused on understanding macrophage heterogeneity in primary and metastatic tumors, less is known about how macrophage diversity contributes to the premalignant niche. To gain insight on myeloid diversity in early mammary lesions, we utilized a transplantable mouse model that progresses through several stages of premalignancy in a predictable timecourse. We hypothesized that we would observe genes that mediate anti-tumor immunity in ductal hyperplasias, and that there would be an expansion and diversification of pro-tumorigenic macrophage populations in lesions undergoing localized invasion. To our surprise, we identified 6 macrophage subpopulations that are very similar in ductal hyperplasias as compared to high grade lesions undergoing localized invasion. Three of these populations are CCR2^+^ (Figure 3), suggesting that 3 subpopulations infiltrate into tumors, and at least one subpopulation (potentially two) is tissue resident. All of these populations express tumor-promoting genes, although two of them resemble macrophages described in the normal mouse mammary gland. Future studies are required to address whether these macrophage subsets differ from those in the normal mammary gland, and whether they are fetal-derived (tissue resident) or bone marrow-derived.

Both tissue resident and infiltrating macrophages have been described in tumors, although less is known about how tissue resident macrophages in primary and metastatic tumors contribute to tumor progression. Zhu et al. showed that embryonically-derived pancreas resident macrophages promote pancreatic ductal adenocarcinoma progression by exerting pro-fibrotic responses (52). In the normal mammary gland, tissue resident macrophages are initially embryonically-derived and function to regulate postnatal mammary gland development and maintain tissue homeostasis (7, 8, 48). Macrophages reside in the adipose stroma or directly adjacent to the ductal epithelium (38, 48, 69). A recent study identified a subpopulation of resident macrophages in the normal mammary gland defined by Lyve1 expression, which associate with ECM-rich regions in the adipose stroma, and function in tissue remodeling (35). In the present study, myeloid cluster 0 largely resembles these stromal macrophages, both of which are defined by high expression of *Lyve1, CD209g, Mrc1*, and *Gas6* (Figure 3). Gene enrichment set analysis shows that endosome and ECM pathways are highly enriched in this cluster, and our co-localization studies show that these macrophages appear to associate with stromal cells surrounding ductal hyperplasias and invasive lesions (Figure 4, Supplementary Figure 6). Genes enriched in cluster 0 are consistent with an alternatively activated/tissue reparative phenotype, and lack a strong inflammatory or antigen presentation signature. Gas6 in particular has strong anti-fibrotic roles in a number of chronic diseases, and primarily functions in the clearance of apoptotic cells during the innate immune response (49, 70). Similarly, Nrp2, which is also highly expressed in cluster 0, was recently shown to facilitate tumor growth by promoting efferocytosis to allow for clearance of apoptotic tumor cells (71).

Myeloid cluster 3 is also enriched for pathways involved in tissue remodeling, including various MMPs and other proteases (Figure 5). We found that these cells remarkably resembled gene expression profiles of tissue resident ductal-associated macrophages (DMs) described in the normal mammary gland, which can intercalate in the ductal epithelial layer and primarily function in tissue remodeling (38, 39). DMs were shown to be initially embryonically-derived, with some turnover from the blood, and persisted in tumors from MMTV-Pymt, MMTV-Neu and MMTV-Wnt transgenic mice. Similar to DMs, cluster 3 highly expresses *Itgax, Cx3cr1*, and *Tmem119*, is negative for *Mrc1*, and shows an enrichment for genes involved in the lysosome, IL-1β signaling, and ECM homeostasis. Unlike the Lyve1^+^ macrophages (MC0), these cells express a number of genes involved in inflammation such as *Cd86, Tnf* and *Il1b*, suggesting the importance in regulating the immune response. Our co-localization studies found a population of CD206^−^ cells within ductal hyperplasias and recruited to the centers of MIN lesions. Notably, areas of necrosis can be detected in expanding high grade MIN PN1a lesions (Figure 1A), and these CD206^−^ cells appear to infiltrate to these regions (Figure 4A, Supplementary Figure 6), supporting the notion of cells from myeloid cluster 3 being recruited to sites of inflammation. The receptor tyrosine kinase Axl, which binds Gas6 and functions in the clearance of phagocytic cells during the innate immune response (70), is also enriched in this population. Interestingly, Axl is overexpressed in human breast cancers and a number of Axl inhibitors are currently in clinical trials (72–74). Although Axl marks classically activated macrophages in innate immunity, Axl inhibition in immune cells was shown to induce an anti-tumor response in mouse models, which was potentiated by PD-1/PD-L1 inhibitors. (75–78). Thus, it is tempting to speculate that Axl^HI^ macrophages have alternative roles in immune suppression, which is supported by enriched expression of Tgfb1 in cluster 3, although functional assays are required to address this idea.

Myeloid cluster 6 shares genes common to both tissue resident macrophages and TAMs (Figure 3A). High expression of *Timd4, Gas6*, and *Mrc1* may suggest that these cells are derived from myeloid cluster 0 or have tissue reparative properties. Indeed it has been suggested that tissue resident macrophages are a source of TAMs (38, 52), although lineage tracing studies are required to address this question. Gene ontology analysis revealed that ERK/MAPK signaling is enriched in cluster 6 (Figure 5), which has been shown to be required for macrophage polarization to an anti-inflammatory/wound healing phenotype (79–81). Likewise, Igf signaling is unique to this subpopulation and has been shown to be secreted by alternatively activated macrophages (57), and Gas6, which inhibits pro-inflammatory cytokines during the innate response, has been shown to stimulate tumor cell invasion by interacting with Axl on adjacent tumor cells (17, 73). Despite these similarities with cluster 0, gene set enrichment analysis defined this subpopulation as inflammatory, exemplified by highly expressed chemokines involved in monocyte or macrophage recruitment to tumors. In addition, numerous pathways involving regulation and activation of T cells are differentially expressed, suggesting an immune-stimulatory phenotype. Interestingly, *Ccl8* is highly enriched in cluster 6, which has been shown to be an important factor for mammary cancer cell dissemination (82), suggesting a potential role in tumor cell invasion. More recently, breast tumor cells induced CCL8 expression in infiltrating TAMs, which in turn induced *Siglec1* and enhanced monocyte recruitment and tumor cell motility (12). In our studies, myeloid cluster 6 strongly expresses *Ccr2*, as well as numerous chemokines such as *Ccl2, Ccl3, Cxcl1*, and *Ccl24* (Figure 5C) that may recruit additional monocytes or macrophages to tumors. Together, these results suggest that myeloid cluster 6 contributes to localized inflammation, recruits other immune cells to tumors, and may ultimately contribute to localized invasion.

While most of the myeloid subpopulations express numerous macrophage markers, cluster 2 was characterized by low expression of *Adgre1* and *CD68*, suggesting that these cells are not fully differentiated. In support, they highly express *Cebpb*, which is found in many myeloid cells and is required for monocyte differentiation (83). This cluster appears to be driven by genes that regulate cell motility, such as *Actb, Anxa2, Tagln2, and Marcks*. Infiltrating macrophages are highly dependent on MARCKS, which regulates actin dynamics and affects cytoskeletal movement (44, 84). Both *Marcks* and *Klf6*, also differentially expressed in cluster 2, modulate inflammation by inducing the secretion of pro-inflammatory factors from neighboring cells (85, 86). Similar to cluster 6, inflammatory chemokines, such as *Ccl8, Ccl7, Ccl2, Ccl3*, and *Ccl24* are differentially expressed, although to a lesser degree to that of cluster 6 (Figure 5). These findings support the notion that these cells are recruited to sites of invasion where they are differentiated into macrophages and contribute to local inflammation.

Our studies identified a number of macrophage subpopulations during the switch to invasive cancer, most of which appear to contribute to local inflammation. These macrophage subpopulations are comprised of a mix of both anti-tumor and pro-tumor genes, and it is feasible to speculate that polarization to a tumor-promoting phenotype is immature. Understanding how these populations contribute to tumor progression will have critical implications for targeting myeloid cells in early and late stage breast cancers. Collectively, our investigation of myeloid cell heterogeneity in the premalignant microenvironment demonstrate a complex balance between cell identity and differential gene expression (Figure 6F), which together serve as a basis for future functional characterization during breast cancer progression.

## Data Availability Statement

The datasets generated for this study can be found in online repositories. The names of the repository/repositories and accession number(s) can be found below: the NCBI BioProject (PRJNA656862).

## Ethics Statement

The animal study was reviewed and approved by Animal Care and Use Committee at Tulane School of Medicine.

## Author Contributions

AI: data curation, data analysis and interpretation, manuscript writing. MM: data analysis and interpretation. ZG: experimental design, data curation. MR: experimental design, data curation, data analysis and interpretation, funding. CB: data curation and data analysis. KS: conceptualizaiton, experimental design, data analysis and interpretation, manuscript editing, funding. CS: data analysis and interpretation, manuscript writing, funding. HM: conceptualization, data analysis and interpretation, manuscript writing, funding. All authors contributed to the article and approved the submitted version.

## Conflict of Interest

The authors declare that the research was conducted in the absence of any commercial or financial relationships that could be construed as a potential conflict of interest.

## Supplementary Material

Supplementary Figure 1 Strategy for defining immune cells in pre-invasive and invasive lesions. **(A)** Quality control filtering to eliminate cell doublets and contamination with mitochondrial DNA. **(B)** Principal component analysis identification of cell outliers at each time point. **(C)** UMAP distribution of immune cells at 8 and 16 weeks post-transplantation, demonstrating poorly represented clusters (filtered out). **(D)** Feature plot depicting *Cd19* mRNA expression, which overlaps with *Cd20* shown in Figure 1D. **(E)** Violin plot demonstrating the distribution of *Cd14* across all clusters. **(F)** Graph depicts the number of cells in each cell type based on *Cd14* (myeloid), *Cd3e* (T cells), *Nkg7* (NK cells), *Cd20* (B cells) mRNA expression.

Supplementary Figure 2 Differential gene expression of myeloid cells. Heatmap representation of the top 10 differentially expressed genes in myeloid clusters 0–10.

Supplementary Figure 3 Classification of myeloid clusters using the ImmGen Databrowser. Box plots show myeloid clusters based on the top 20 differentially expressed genes: dendritic cells (DC), macrophages (MF), monocytes (MO), granulocytes (GN), and mast cells (MC).

Supplementary Figure 4 Characteristics of myeloid clusters 5 and 7. **(A)** Violin plots for *MKi67, Pclaf* and *Stmn1*, and UMAP illustrating cells in different stages of the cell cycle show that myeloid cluster 5 is a proliferating cell population. **(B)** Violin plots depict *Cxcr2, Il1b*, and *Cebpb* expression in myeloid clusters 0–10.

Supplementary Figure 5 Gene expression across myeloid clusters for selected genes. **(A)** Violin plot for *Cd274* (PD-L1) across myeloid clusters 0–10. **(B)** Feature plots for *Cd74, Ms4a7* and *Hexb* show distribution of expression across myeloid clusters.

Supplementary Figure 6 Quantitation of putative macrophage markers. Graphs depict the number CSFR1^+^ cells expressing **(A)** Lyve-1 or Gas6, or **(B)** CD206, located within ductal regions (“within lesion”) or regions surrounding the hyperplastic/MIN cells (“surrounding lesion”). **(C)** Images depict immunofluorescent co-staining using antibodies to CSFR1 (green) or CD206 (red) and stained with DAPI of PN1a established tumors (# of mice). White arrows: CSFR1^+^CD206^+^; green arrows: CSFR1^+^CD206^−^; red arrows: CSFR1^−^CD206^+^. Scale bars = 100 and 25 μm for inset. Graph shows the quantitation of CD206^+^ or CD206^−^ CSFR1^+^ cells within tumors. All graphs show the number of cells per field of view (FOV) after counting 10 random FOVs ± SEM (*n* = 3 per timepoint). Two way ANOVA was performed to determine statistical differences between groups. ^*^*p* = 0.05, ^**^*p* = 0.001, ^****^*p* < 0.0001.

Supplementary Table 1 Top 20 differentially expressed genes (DEGs) across macrophage populations (MC0, MC2, MC3, MC4, MC6, MC8).

Supplementary Table 2 Gene set enrichment analysis (GSEA) for selected macrophage populations. Differentially expressed genes across pairs of analyzed myeloid clusters were uploaded into GSEA for the identification of enriched pathways, as demonstrated in Figures 5, 6.

Supplementary Table 3 Significant Gene Ontology terms in from GSEA analysis of selected macrophage clusters.

Supplementary Table 4 Analysis of cluster-specific pathways. *p*-value defines the significance of enrichment for genes in each cluster in each pathway.

Supplementary Table 5 Analysis of enriched pathways and their distribution across time points. *p*-value defines the significance of enrichment for genes in each cluster in each pathway.

Supplementary Table 6 Top 50 differentially expressed genes in pre-invasive as compared to invasive lesions for selected macrophage clusters.
