# Supplementary figures and images for: Diverse Macrophage Populations Contribute to the Inflammatory Microenvironment in Premalignant Lesions During Localized Invasion
Source: Front Oncol. 2020 Sep 24;10:569985. doi: 10.3389/fonc.2020.569985 (PMC7541939; doi:10.3389/fonc.2020.569985)

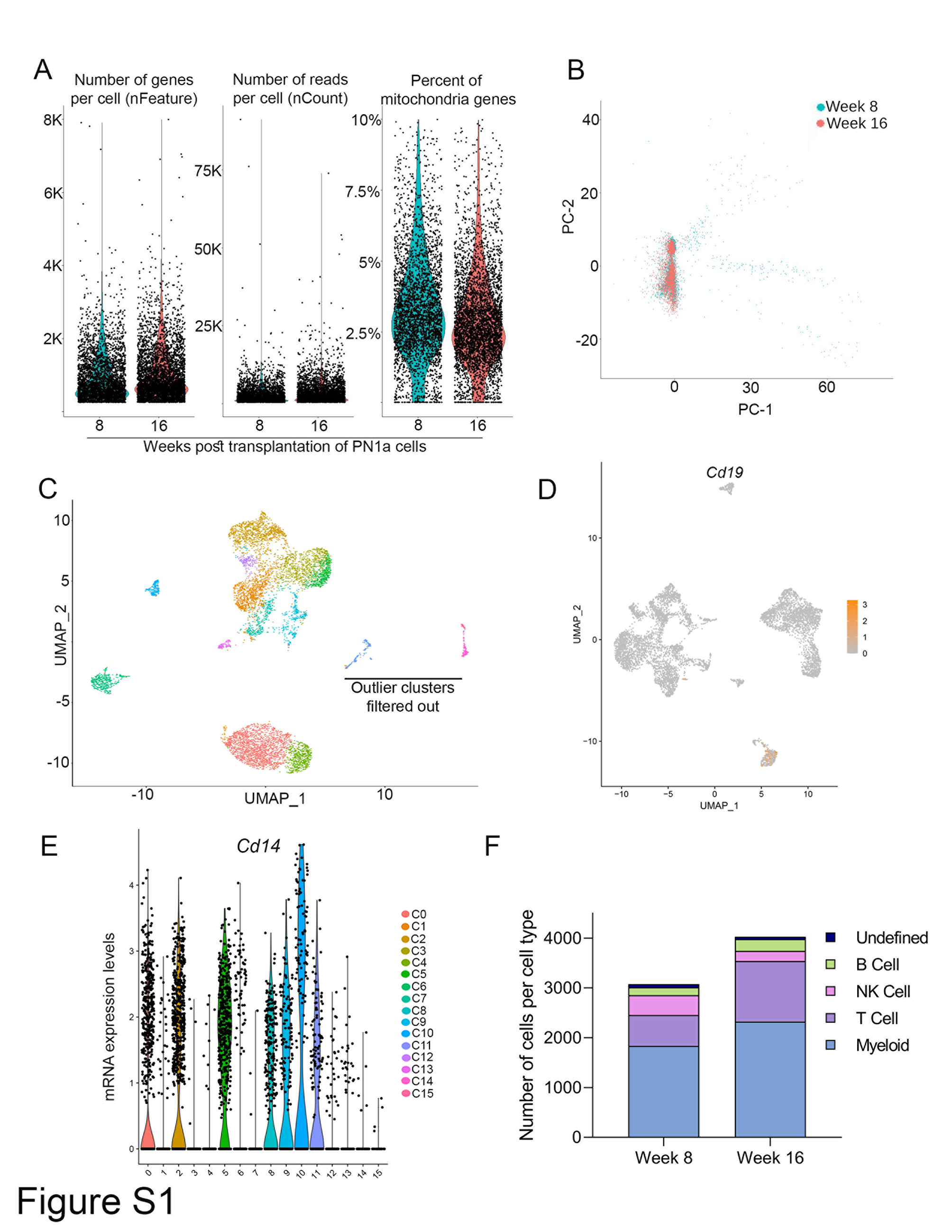

Supplement: Supplementary Figure 1 — Strategy for defining immune cells in pre-invasive and invasive lesions. (A) Quality control filtering to eliminate cell doublets and contamination with mitochondrial DNA. (B) Principal component analysis identification of cell outliers at each time point. (C) UMAP distribution of immune cells at 8 and 16 weeks post-transplantation, demonstrating poorly represented clusters (filtered out). (D) Feature plot depicting Cd19 mRNA expression, which overlaps with Cd20 shown in Figure 1D. (E) Violin plot demonstrating the distribution of Cd14 across all clusters. (F) Graph depicts the number of cells in each cell type based on Cd14 (myeloid), Cd3e (T cells), Nkg7 (NK cells), Cd20 (B cells) mRNA expression. [file Image_1.TIF]

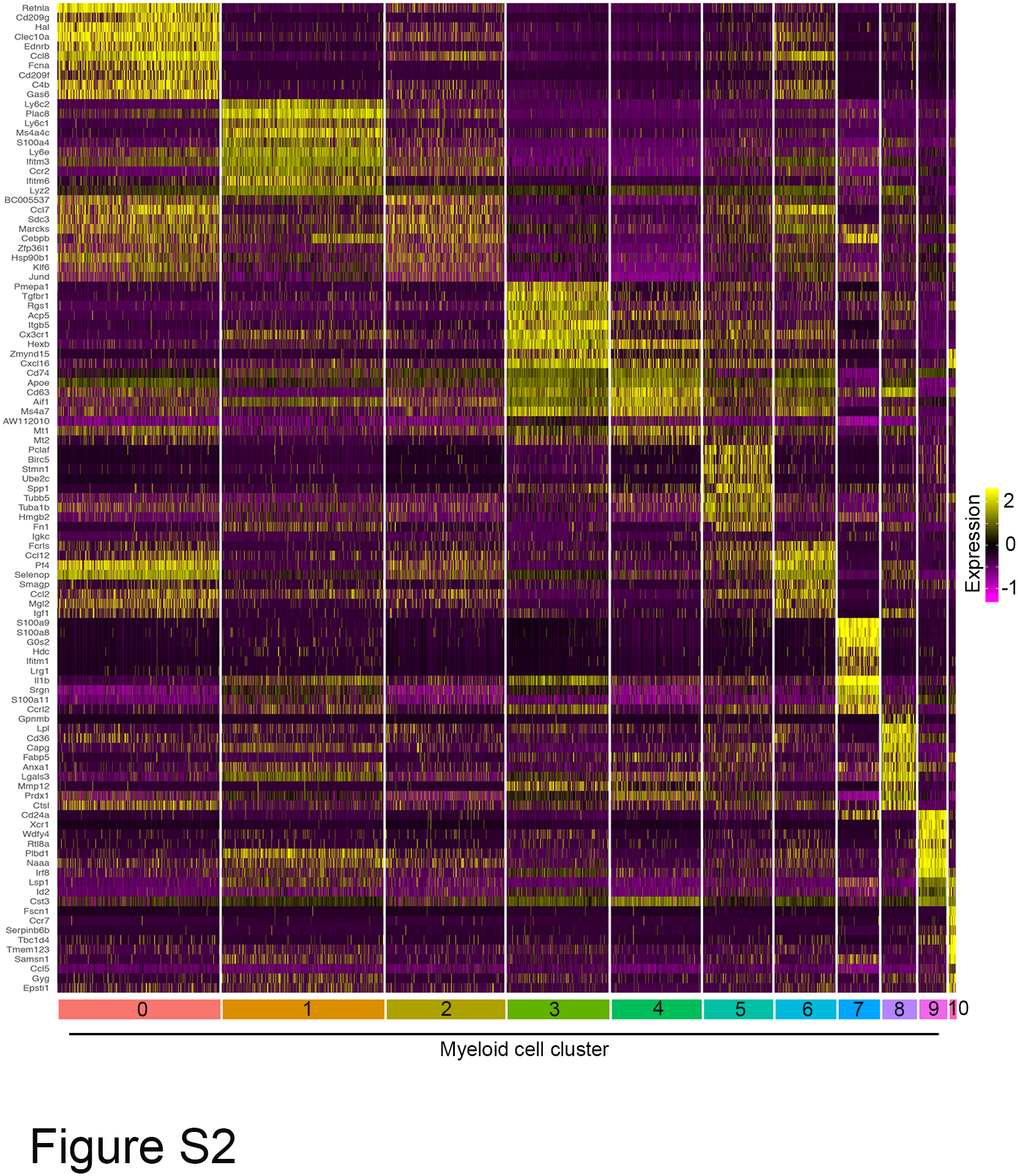

Supplement: Supplementary Figure 2 — Differential gene expression of myeloid cells. Heatmap representation of the top 10 differentially expressed genes in myeloid clusters 0–10. [file Image_2.TIF]

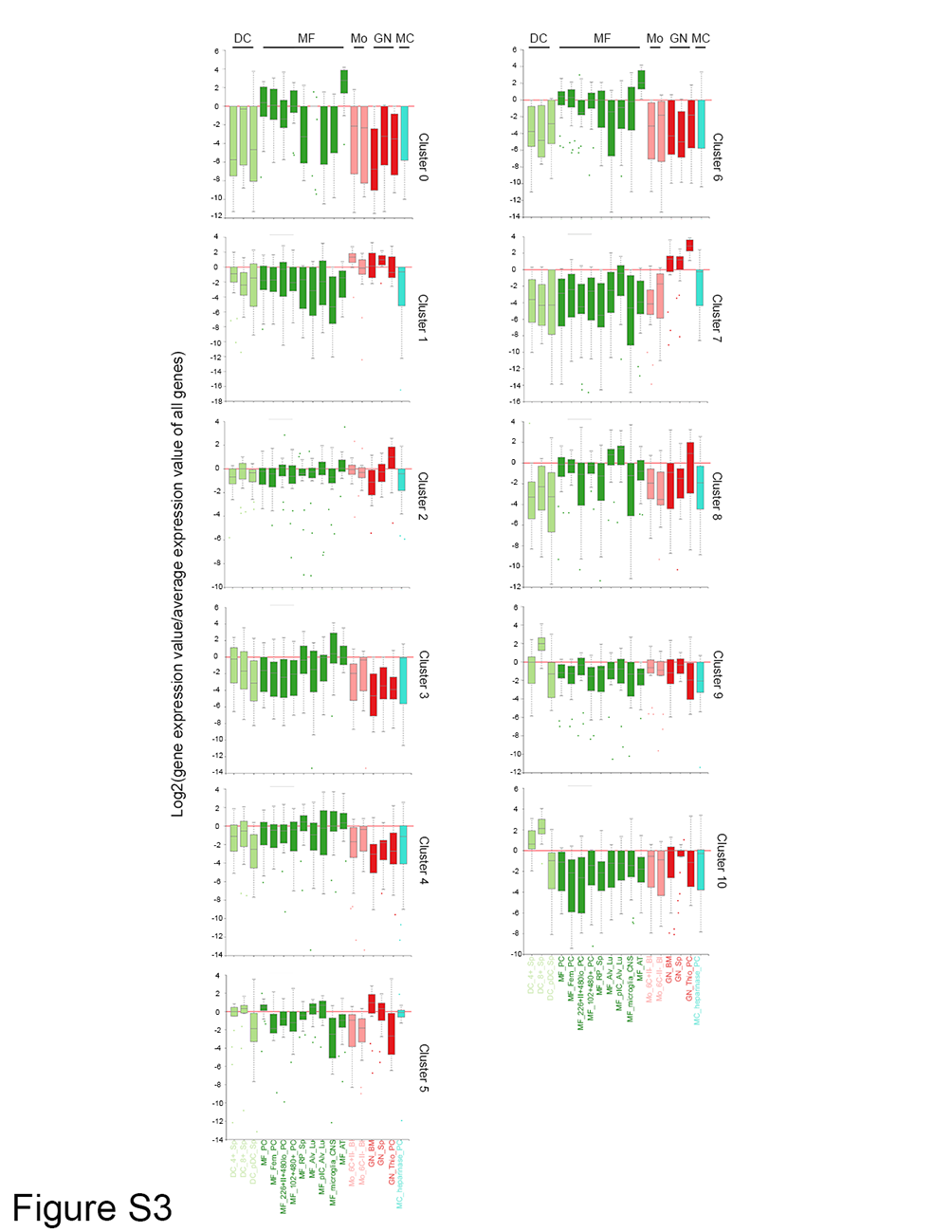

Supplement: Supplementary Figure 3 — Classification of myeloid clusters using the ImmGen Databrowser. Box plots show myeloid clusters based on the top 20 differentially expressed genes: dendritic cells (DC), macrophages (MF), monocytes (MO), granulocytes (GN), and mast cells (MC). [file Image_3.TIF]

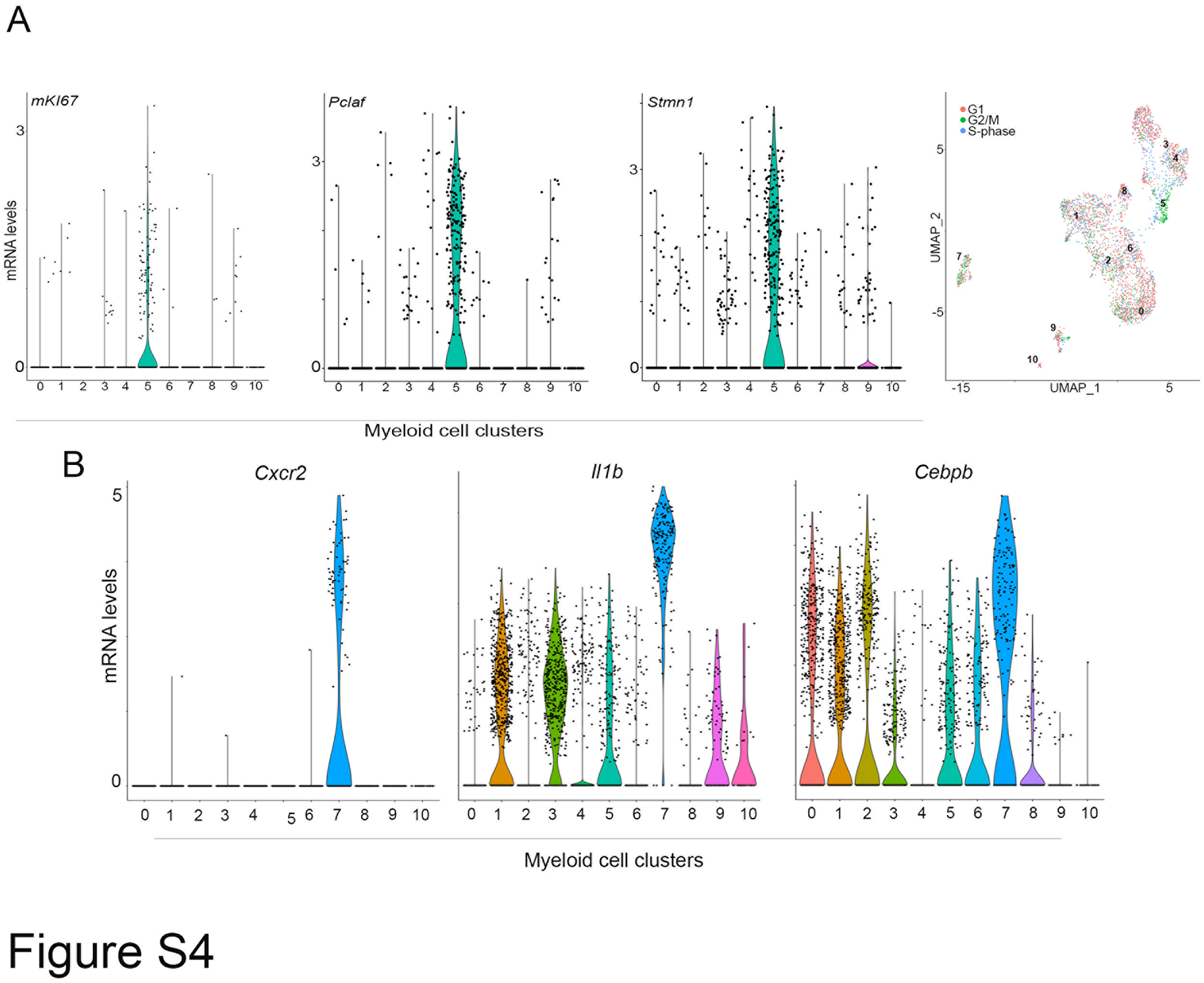

Supplement: Supplementary Figure 4 — Characteristics of myeloid clusters 5 and 7. (A) Violin plots for MKi67, Pclaf and Stmn1, and UMAP illustrating cells in different stages of the cell cycle show that myeloid cluster 5 is a proliferating cell population. (B) Violin plots depict Cxcr2, Il1b, and Cebpb expression in myeloid clusters 0–10. [file Image_4.TIF]

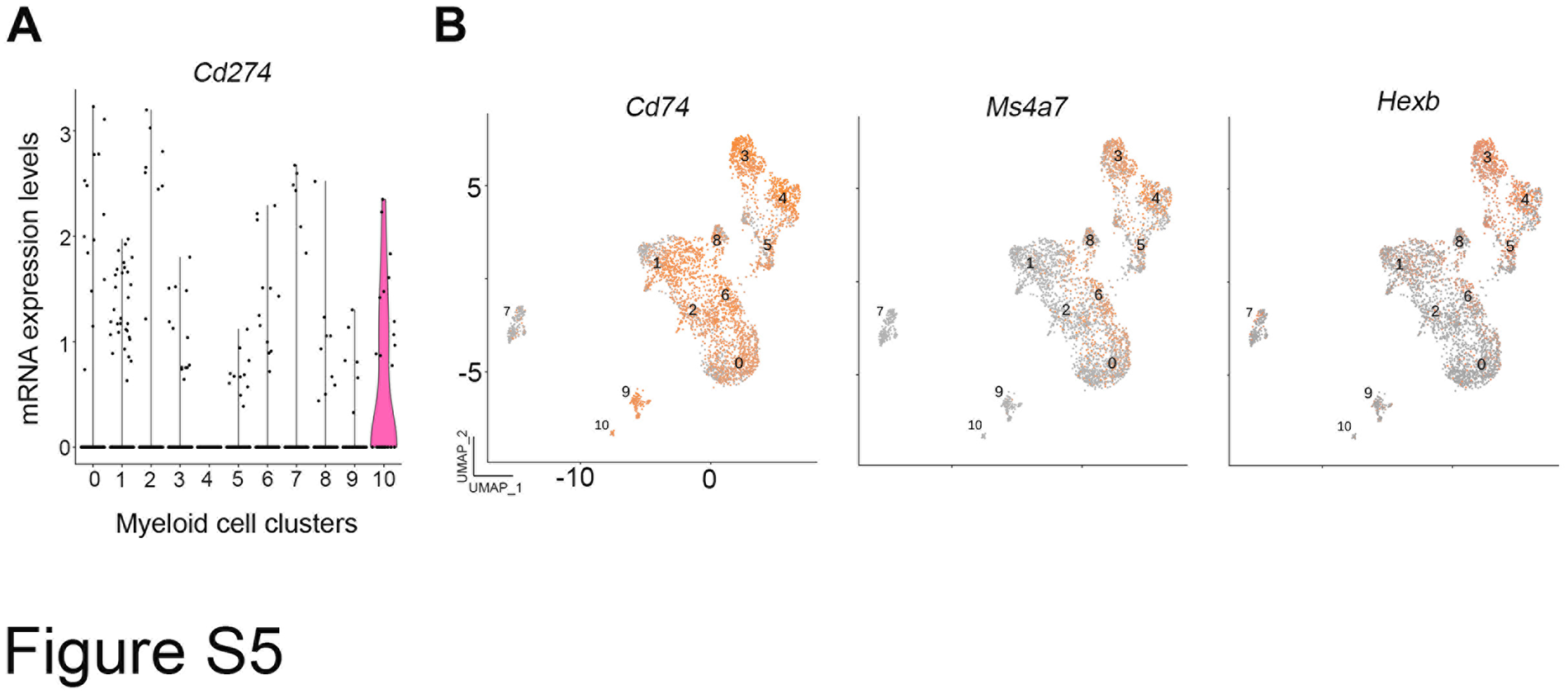

Supplement: Supplementary Figure 5 — Gene expression across myeloid clusters for selected genes. (A) Violin plot for Cd274 (PD-L1) across myeloid clusters 0–10. (B) Feature plots for Cd74, Ms4a7 and Hexb show distribution of expression across myeloid clusters. [file Image_5.TIF]

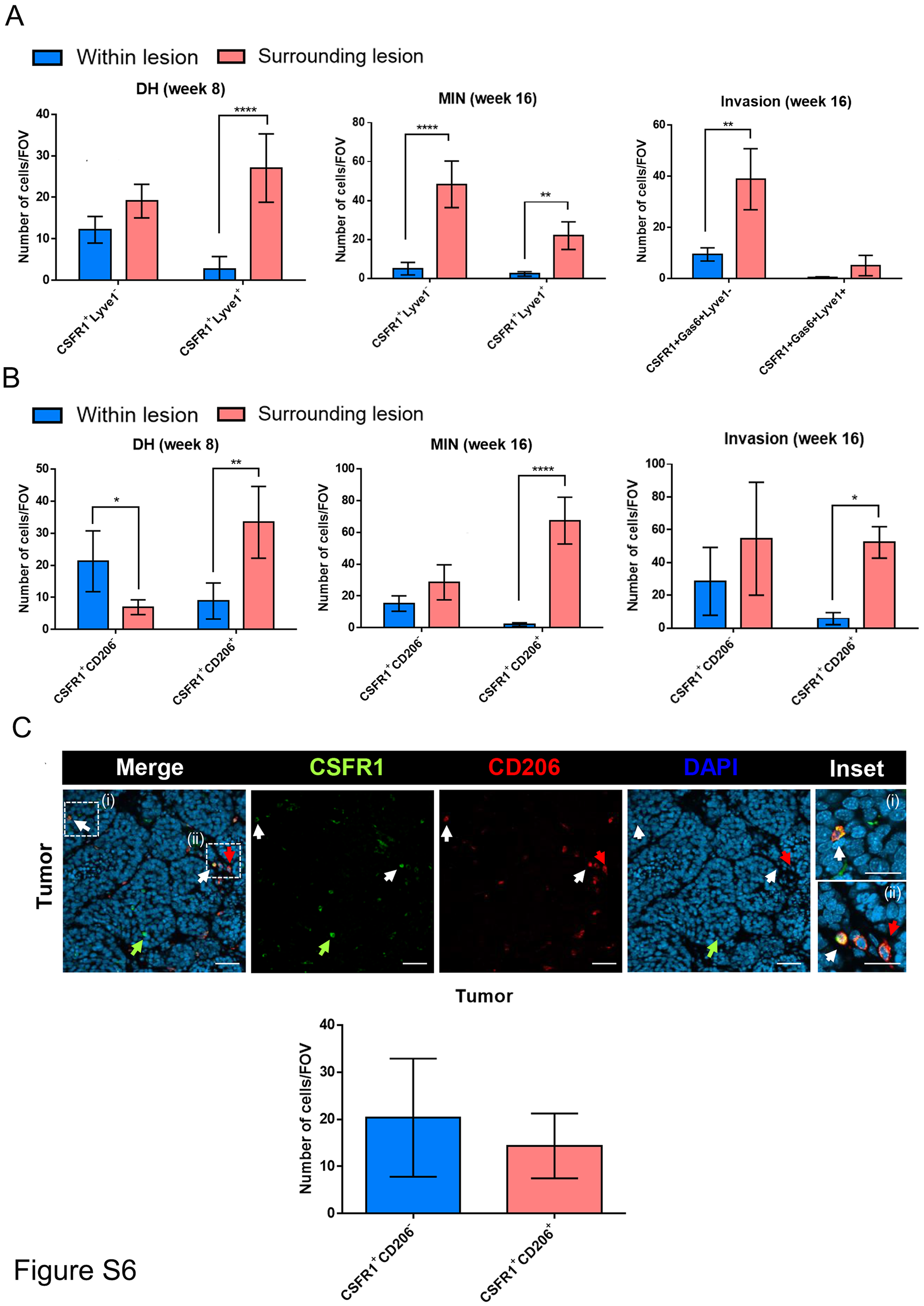

Supplement: Supplementary Figure 6 — Quantitation of putative macrophage markers. Graphs depict the number CSFR1+ cells expressing (A) Lyve-1 or Gas6, or (B) CD206, located within ductal regions (“within lesion”) or regions surrounding the hyperplastic/MIN cells (“surrounding lesion”). (C) Images depict immunofluorescent co-staining using antibodies to CSFR1 (green) or CD206 (red) and stained with DAPI of PN1a established tumors (# of mice). White arrows: CSFR1+CD206+; green arrows: CSFR1+CD206−; red arrows: CSFR1−CD206+. Scale bars = 100 and 25 μm for inset. Graph shows the quantitation of CD206+ or CD206− CSFR1+ cells within tumors. All graphs show the number of cells per field of view (FOV) after counting 10 random FOVs ± SEM (n = 3 per timepoint). Two way ANOVA was performed to determine statistical differences between groups. *p = 0.05, **p = 0.001, ****p < 0.0001. [file Image_6.TIFF]
