# Supplementary material for: Diverse Macrophage Populations Contribute to the Inflammatory Microenvironment in Premalignant Lesions During Localized Invasion
Source: Front Oncol. 2020 Sep 24;10:569985. doi: 10.3389/fonc.2020.569985 (PMC7541939; doi:10.3389/fonc.2020.569985)
Supplement: Supplementary Table 4 — Analysis of cluster-specific pathways. p-value defines the significance of enrichment for genes in each cluster in each pathway. [file Table_4.DOCX]

**Table S4.** **Analysis of cluster-specific pathways.** p value defines the significance of enrichment for genes in each cluster in each pathway.

| **Pathway** | **p value** | | | | | |
| --- | --- | --- | --- | --- | --- | --- |
|  | **Week 8 (pre-invasive)** | | | **Week 16 (invasive)** | | |
|  | **MC 3** | **MC 4** | **MC 8** | **MC 0** | **MC 2** | **MC 6** |
| Focal adhesion-PI3K-Akt-mTOR signaling pathway (*Ifnar2, Gngt2, Itgb5, Lpar6, Itgax, Pdgfa, Itgav, creb5*) | 2.04E-02 |  |  |  |  |  |
| Matrix metalloproteinases  (*Mmp12, Mmp14*) | 3.64E-02 |  |  |  |  |  |
| Oxidative damage  *(C1qb, C1qa, C1qc)* |  | 2.56E-03 |  |  |  |  |
| PPAR signaling pathway  (*Slc27a1, Fabp4, Fabp5, Lol, Dbi, Cd36*) |  |  | 7.37E-04 |  |  |  |
| Retinol metabolism  (*Lpl, Cd36, Dhrs3*) |  |  | 1.49E-02 |  |  |  |
| Glutathione metabolism  (*Gpx1, Gpx4, Idh1, Anpep*) |  |  | 1.25E-04 |  |  |  |
| Keap1-Nrf2  (*Cebpb, Maf, Hmox1*) |  |  |  | 2.20E-0.4 |  |  |
| Delta-notch signaling pathway  (*App, Jun, Rbpj)* |  |  |  | 3.58E-02 |  |  |
| Alpha6-beta4 integrin signaling pathway  (*Rpsa, Ywhaz*) |  |  |  |  | 2.95E-02 |  |
| Statin pathway  (*Abca1, Apoe1, Pltp*) |  |  |  |  |  | 1.95E-04 |
| Factors/pathways affecting insulin-like growth factor-Akt signaling (*Igfbp4, Igf1*) |  |  |  |  |  | 1.51E-02 |
