# Supplementary material for: Diverse Macrophage Populations Contribute to the Inflammatory Microenvironment in Premalignant Lesions During Localized Invasion
Source: Front Oncol. 2020 Sep 24;10:569985. doi: 10.3389/fonc.2020.569985 (PMC7541939; doi:10.3389/fonc.2020.569985)
Supplement: Supplementary Table 5 — Analysis of enriched pathways and their distribution across time points. p-value defines the significance of enrichment for genes in each cluster in each pathway. [file Table_5.DOCX]

**Table S5.** **Analysis of enriched pathways and their distribution across time points.** p value defines the significance of enrichment for genes in each cluster in each pathway.

| **Pathway** | | **p value** | | | | | |
| --- | --- | --- | --- | --- | --- | --- | --- |
|  |  | **Week 8 (pre-invasive)** | | | **Week 16 (invasive)** | | |
|  |  | **MC 3** | **MC4** | **MC 8** | **MC 0** | **MC 2** | **MC 6** |
| **Week 8,16** | Chemokine signaling pathway | 8.64E-04 |  | 1.38E-02 | 4.43E-05 | 9.92E-04 | 1.59E-04 |
|  | Microglia pathogen phagocytosis pathway | 3.91E-06 | 6.67E-06 | 1.71E-02 |  |  | 1.09E-04 |
|  | p53 signaling |  |  |  |  |  |  |
|  | Id signaling pathway | 1.60E-02 |  |  |  |  | 3.82E-02 |
|  | TNFα NF-κB signaling pathway |  | 7.57E-03 |  | 3.91E-02 |  |  |
| **Week 8** | Macrophage markers | 2.27E-06 | 1.82E-03 | 7.39E-03 |  |  |  |
|  | Inflammatory response pathway |  |  | 2.70E-04 |  |  |  |
|  | Folic acid network |  |  | 2.22E-04 |  |  |  |
| **Week 16** | MAPK signaling pathway |  |  |  | 4.92E-04 | 3.81E-03 | 1.59E-02 |
|  | EGFR1 signaling pathway |  |  |  | 9.95E-04 | 3.46E-02 | 2.31E-02 |
|  | TGFβ1 signaling pathway | 2.08E-03 |  |  | 1.08E-03 | 1.84E-02 | 3.85E-03 |
|  | Novel Jun-Dmp1 pathway |  |  |  | 1.45E-03 |  | 1.07E-02 |
|  | Il-6 signaling pathway |  |  |  | 6.70E-04 |  |  |
|  | Il-3 signaling pathway |  |  |  |  | 7.54E-03 |  |
